# MiR-505 suppressed the growth of hepatocellular carcinoma cells via targeting IGF-1R

**DOI:** 10.1042/BSR20182442

**Published:** 2019-07-02

**Authors:** Liang Ren, Yongshan Yao, Yang Wang, Shengqiang Wang

**Affiliations:** 1 Department of Ultrasound and Imaging, Yichang Yiling Hospital, Yichang city 443100, Hubei province, China; 2 Emergency and Trauma Surgery, The First College of Clinical Medical Science, China Three Gorges University, Yichang city 443100, Hubei province, China; 3 Department of Pediatrics, Yichang Yiling Hospital, No.32 of Dong Hu street, Yiling district, Yichang city 443100, Hubei province, China

**Keywords:** AKT, GLUT1, glycolysis, Hepatocellular carcinoma, IGF-1R, miR-505

## Abstract

Hepatocellular carcinoma (HCC) is one of the most common cancers globally. An increasing body of evidence has demonstrated the critical function of microRNAs (miRNAs) in the initiation and progression of human cancers. Here, we showed that miR-505 was down-regulated in HCC tissues and cell lines. Reduced expression of miR-505 was significantly correlated with the worse prognosis of HCC patients. Overexpression of miR-505 suppressed the proliferation, colony formation and induced apoptosis of both HepG2 and Huh7 cells. Further mechanism study uncovered that miR-505 bound the 3′-untranslated region (3′-UTR) of the insulin growth factor receptor (IGF-1R) and inhibited the expression of IGF-1R in HCC cells. The down-regulation of IGF-1R by miR-505 further suppressed the phosphorylation of AKT at the amino acid S473. Consistently, the abundance of glucose transporter (GLUT) 1 (GLUT1) was reduced with the overexpression of miR-505. Down-regulation of GLUT1 by miR-505 consequently attenuated the glucose uptake, lactate production and ATP generation of HCC cells. Collectively, our results demonstrated the tumor suppressive function of miR-505 possibly via inhibiting the glycolysis of HCC cells. These findings suggested miR-505 as an interesting target for designing anti-cancer strategy in HCC.

## Introduction

Hepatocellular carcinoma (HCC) is one of the most common malignancies with high mortality and occurrence around the world [1–3]. Early diagnosis of HCC is particularly difficult due to the lack of efficient biomarkers. Most of HCC patients are diagnosed as advanced stage, which is usually accompanied by distant metastasis [4]. Chemotherapeutic interventions and liver transplantation have greatly improved the outcome of HCC patients, however, the long-term survival of HCC patients still remains poor [4]. Therefore, it is urgent to identify novel factors and characterize the underlying mechanisms involved in the progression of HCC.

MicroRNAs (miRNAs) are a class of evolutionarily conserved, single-stranded, non-coding RNA molecules with an approximate length of 18–24 nucleotides [5,6]. MiRNAs play important roles in regulating gene expression by pairing with the 3′-untranslated region (3′-UTR) of target mRNAs, which results in their degradation or translation inhibition [7]. Increasing evidence has demonstrated that miRNAs regulate cell proliferation, differentiation and respond to stress conditions [6,8]. Dysfunction of miRNAs was associated with the formation and progression of human diseases, especially cancers [9–12]. Interestingly, many miRNAs are aberrantly expressed in HCC and play important roles in the initiation and progression of cancer. For example, miR-1468 promoted HCC development via activating AKT signaling [13]. Additionally, miR-199 acted as a tumor suppressor in HCC by down-regulating the expression of regulators of G-protein signaling 17 [14]. To get a whole picture of the expression of miRNAs in HCC, we performed miRNA-seq to screen the miRNAs with aberrant expression in HCC tissues compared with that of corresponding normal tissues. Our results indicated the significant down-regulation of miR-505 in HCC tissues compared with normal controls (data not shown). It has been found that miR-505 suppressed the proliferation, migration and invasion of osteosarcoma cells [15]. The potential tumor suppressive function of miR-505 was also demonstrated in cervical cancer and endometrial cancer [16,17]. Recent studies showed that miR-505 enhanced docorubicin-induced cytotoxicity, suppressed HCC cell growth via targeting HMGB1 [18,19]. However, the functional mechanisms of miR-505 in HCC have not been fully investigated so far.

The Warburg effect, also known as aerobic glycolysis, has been considered as the hallmark of cancer cells. Cancer cells metabolize glucose via glycolysis but not mitochondrial respiratory even in the presence of enough oxygen [20,21]. To meet with the need of rapid cell growth, the key regulators of glycolysis, such as glucose transporters (GLUTs) and glycolytic enzymes, are usually overexpressed in cancer cells. It has been documented that insulin stimulated glucose metabolism via insulin growth factor receptor (IGF-1R), consequently activating the phosphorylation of AKT and up-regulated the expression of GLUTs [22–24]. IGF-1R and AKT-GLUT1 axis might be promising targets of miRNAs to affect the progression of cancers.

In the present study, we found that miR-505 was significantly decreased in HCC tissues and associated with the poor prognostic features of HCC patients. Overexpressed miR-505 inhibited the malignant behaviors of HCC cells via targeting IGF-1R, which finally suppressed the glycolysis of HCC cells.

## Materials and methods

### Clinical tissues

Sixty paired HCC tissues and adjacent normal tissues were obtained from HCC patients via surgical resection at Yichang Yiling Hospital during January 2010 to October 2011. None of these patients received radiotherapy or chemotherapy prior to the surgery. Tissues were frozen in liquid nitrogen before the experiments. Informed consents were signed and obtained from all patients. The experiments were approved by the Ethical Committee of the Yichang Yiling Hospital and complied with the World Medical Association Declaration of Helsinki. The grade and stage of liver cancer was evaluated according to the criteria of UICC/AJCC, 2010. The clinical characteristics of enrolled patients were summarized in Table 1.

**Table 1:** The correlation between the expression of miR-505 and the clinical features of HCC patients

Clinical features	Number	High-miR-505	Low-miR-505	*P*-value
**Age, years**				
≤60	20	4	16	*P*>0.05
>60	40	15	25	
**Gender**				
Male	26	8	18	*P*>0.05
Female	34	11	23	
**Tumor size (cm)**				
≤4	30	15	15	*P*<0.001
>4	30	4	26	
**Histological grade**				
High	27	16	11	*P*<0.001
Poor	33	3	30	
**Lymph node metastasis**				
Negative	28	14	14	*P*<0.001
Positive	32	5	27	
**TNM stage**				
I-II	24	15	9	*P*<0.001
III-IV	36	4	32	

### Cell culture and transfection

HCC cell lines including HepG2, SMMC-7721, Bel-7404 and Huh7 were purchased from the Cell Bank of the Chinese Academy of Sciences (Shanghai, China). Cells were cultured in RPMI-1640 medium (Gibco, Grand Island, NY, U.S.A.) with 10% fetal bovine serum (FBS, Gibco, Grand Island, NY, U.S.A.) at 37°C in a 5% CO_2_ atmosphere. For cell transfection, the corresponding miRNA was transfected into HCC cells at the final concentration of 20 μM with Lipofectamine 3000 (Thermo Fisher Scientific, U.S.A.) as described [25,26].

### Determination of cell proliferation

The proliferation of both HepG2 and Huh7 cells was determined using Cell Counting Kit-8 (CCK-8) (Beyotime, Shanghai, China) according to the manufacturer’s protocol. Briefly, HCC cells transfected with control miRNA or miR-505 mimics were seeded in 96-well plates with approximately 1000 cells per well. With the interval of 24 h, 10 μl of CCK-8 reagent was added into the medium and incubated at 37°C for 3 h. The absorbance of each well at 450 nm was measured with the microplate reader (Bio-Rad Laboratories, Inc., Hercules, CA, U.S.A.). The assay was performed in triplicates.

### Colony formation assay

Both HepG2 and Huh7 cells transfected with control miRNA or miR-505 mimics were seeded in 35-mm plate with the density of 3000 cells in the medium containing 10% FBS. After 2 weeks, colonies were fixed with 4% paraformaldehyde and then stained with 1% Crystal Violet at room temperature (RT) for 15 min. The colony number was counted with the microscope.

### MiRNA extraction and quantitative real-time PCR

MiRNA was extracted with the miRcute miRNA isolation kit (Tiangen, Beijing, China) according to the protocol. Briefly, 1 ml of MZ lysis buffer was added into the cells, pipetted several times and incubated at RT for 5 min. Two hundred microliters of chloroform was added into the samples, vortexed for 15 s and kept for 5 min. Samples were centrifuged at 12000 rpm for 15 min at 4°C and the upper layer was collected; 1.5 volume of ethanol was added and the samples were transferred into the miRspin column to centrifuge for 15 s. Five hundred microliters of MRD buffer was added into the column, incubated for 2 min at RT and then spinned at 12000 rpm for 30 s. And then, the column was washed twice by adding 500 μl of washing buffer into columns and centrifuged at 12000 rpm for 30 s. Fifty microliters of RNase-free water was added and RNA was eluted by centrifuging at 12000 rpm for 2 min. The concentration and quantity of RNA was determined with Nano-Drop 2000 (Thermo Fisher Scientific, U.S.A.). cDNA was synthesized with the miRcute miRNA First-Strand cDNA Synthesis Kit (Tiangen, Beijing, China). Quantitative PCR was performed using the SYBR Green Mix (Bio-Rad Laboratories, Inc., Hercules, CA, U.S.A.) with the platform of CFX96 system (Bio-Rad Laboratories, Inc., Hercules, CA, U.S.A.). PCR conditions were as below: 95°C for 5 min; 40 cycles at 95°C for 10 s and 60°C for 1 min. Expression of U6 RNA was detected for normalization. The relative expression of miR-505 was calculated with 2^−ΔΔ*C*^_T_ method. Primers of miR-505 and U6 were provided as (Table 2).

**Table 2:** Multivariate Cox regression of prognostic factors for the overall survival of HCC patients

	Univariate analysis			Multivariate analysis		
Parameter	HR	95% CI	*P*	HR	95% CI	*P*
Age (≤60 vs. >60)	0.610	0.37–1.02	0.056			
Gender (Male vs. Female)	0.723	0.45–0.96	0.062			
Tumor size (≤4 vs. >4)	2.062	1.24–3.08	0.001	0.384	0.126–2.080	0.315
Lymph node metastasis (Negative vs. Positive)	2.434	1.54–4.18	<0.001	2.126	0.524–8.871	0.282
TNM stage (I-II vs. III-IV)	2.122	1.34–3.89	<0.001	2.151	0.923–5.187	0.072
MiR-505 expression (Low vs. High)	3.166	1.86–4.20	<0.001	2.684	1.782–4.822	<0.001

### Targets prediction

The database TargetMiner (https://www.isical.ac.in/∼bioinfo_miu/final_html_targetminer/hsa-miR-505-3p.html) was used to predict the targets of miR-505. Briefly, input the name of ‘miR-505’ into the database and select hsa-miR-505. Click the link of ‘TargetMiner’ to obtain the predicted targets of miR-505.

### Western blot

Both HepG2 and Huh7 cells were transfected with control miRNA or miR-505 mimics. After transfection for 48 h, cells were harvested and lyzed with the RIPA buffer (Beyotime, Shanghai, China) on ice for 10 min. The protein concentration was determined via the BCA protein assay (Beyotime, Shanghai, China). Equal amount of protein was separated by SDS/PAGE and transferred on to the nitrocellulose membranes (Millipore, Bedford, MA, U.S.A.). After blocking with 5% non-fat milk for 1 h at RT, membrane was incubated with the primary antibody (anti-IGF-1R (9750S), anti-pAKT (Ser^473^, 9271S), anti-AKT (9272S), Cell Signaling Technology, Danvers, MA, U.S.A.; anti-GLUT1 (sc-377228), Santa Cruz Biotechnology (Dallas, TX, U.S.A.) for 2 h at RT. Afterward, membrane was incubated with horseradish peroxidase (HRP)–conjugated secondary antibody (1:3000, Beijing Zhongshan Golden Bridge Biotechnology Co., Ltd., Beijing, China) for 1 h at RT. Protein bands were visualized with Enhanced ECL detection kit (Millipore, Bedford, MA, U.S.A.).

### Flow cytometry analysis for cell apoptosis

Cell apoptosis was determined with the Dead Cell Apoptosis Kit containing annexin V-fluorescein isothiocyanate (FITC) and propidium iodide (PI) (V13242, Thermo Fisher Scientific, U.S.A.). Briefly, HCC cells expressing miRNA control or miR-505 mimics were harvested after transfection for 48 h. Cells were washed twice with pre-cold PBS and centrifuged at 3000×***g*** for 5 min. Cells were resuspended to approximately 1 × 10^6^ cells/ml with the 1× annexin-binding buffer. One hundred microliters of cell suspension was then stained with Annexin V FITC and PI for 15 min in the darkness at RT. Cell apoptosis was analyzed with the flow cytometer (FACS, Beckman Coulter, Inc.).

### Luciferase reporter assay

Wild-type or mutated 3′-UTR of IGF-1R containing the putative binding site of miR-505 was constructed into the luciferase reporter vector psiCHECK-2. Both HepG2 and Huh7 cells were cultured in 24-well plate and co-transfected with either wild-type or mutated luciferase vector of IGF-1R with miR-505 mimics or control miRNA. After transfection for 48 h, cells were harvested and the luciferase reporter activity was determined by the Dual-GLO Luciferase Assay System (Promega Corporation, Madison, WI, U.S.A.). The experiment was performed in triplicates. Relative luciferase activity was normalized to the activity of *Renilla*.

### Glucose uptake assay

Glucose uptake of HepG2 and Huh7 cells was determined with the Glucose Uptake Colorimetric Assay kit (BioVision, Milpitas, CA, U.S.A.) according to the manufacturer’s instructions. HCC cells transfected with miRNA control or miR-505 mimics were seeded in 96-well plate and starved with the Krebs–Ringer–Phosphate–HEPES buffer containing 2% BSA for 30 min. Afterward, 10 mM 2-DG was added and incubated for 20 min. Medium was removed and cells were washed twice with pre-cooled PBS. Cells were resuspended with 3 ml of 10 mM Tris/HCl buffer (pH 8.0) and disrupted with the microtip sonicator. After heating at 80°C for 15 min, cell lysates were centrifuged at 15000×***g*** for 20 min at 4°C. Supernatant was collected and glucose uptake was determined with the kit according to the protocol.

### Determination of the lactate production

HepG2 and Huh7 cells transfected with indicated miRNA were cultured in 96-well plate for 48 h. Lactate production was determined using the Lactate Assay Kit (MAK064, Sigma–Aldrich, U.S.A.) according to the manufacturer’s protocol. Briefly, cells were harvested and homogenized with the lactate assay buffer on ice. After centrifuging at 13000×***g*** for 10 min at 4°C, 50 μl of supernatant was transferred into a new 96-well plate. Fifty microliters of master reaction mix was added and incubated at RT for 30 min. Meanwhile, standard solution was prepared by diluting 0-, 2-, 4-, 6-, 8- and 10 μl of lactate standard reagent into 96-well plate. Absorbance of each well at 570 nm was measured with microplate reader (Bio-Rad, Laboratories, Inc., Hercules, CA, U.S.A.).

### Statistical analysis

Results were shown as mean ± standard deviation from three independent experiments. Differences between groups were determined by Student’s *t* test or one way-ANOVA using the SPSS 17.0 (La Jolla, U.S.A.) statistical software package. *P*-values less than 0.05 were considered to be statistically significant.

## Results

### The expression of miR-505 was decreased in HCC tissues

**Figure 1:**
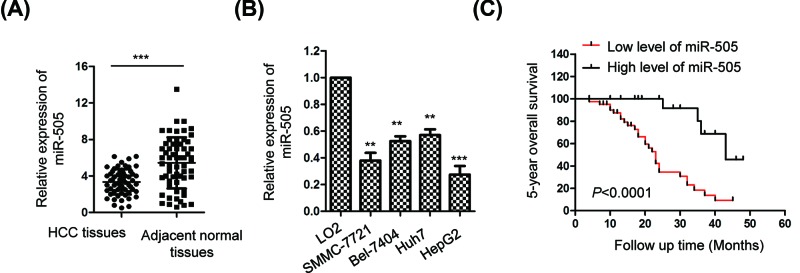
MiR-505 was down-regulated in HCC tissues and cell lines (**A**) The expression of miR-505 was detected in 60 paired HCC tissues and adjacent normal tissues. (**B**) RT-qPCR analysis was performed to compare the level of miR-505 in HCC cell lines (SMMC-7721, Bel-7404, Huh7, HepG2) and normal hepatic cell line LO2. (**C**) The correlation between the expression of miR-505 with the overall survival of HCC patients was analyzed with the log rank test. **P<0.01; ***P<0.001

As our previous primary data showed the decreased expression of miR-505 in HCC, to further confirm this, the level of miR-505 was detected by RT-qPCR analysis in 60 paired of randomly selected tumor tissues and corresponding adjacent normal tissues regardless of the age, gender, nationalities and region of HCC patients. The data showed that miR-505 was significantly down-regulated in HCC tissues in comparison with that of the matched non-tumor tissues (Figure 1A). To further validate the decreased expression of miR-505 in HCC, the expression of miR-505 in HCC cell lines and normal cell LO2 was detected. Compared with the normal hepatic cell LO2, miR-505 was significantly down-regulated in HCC cell lines including HepG2, SMMC-7721, Bel-7404 and Huh7 (Figure 1B). To illustrate the correlation between the expression of miR-505 and the clinical characteristics of HCC patients, all the 60 HCC patients were divided into miR-505-high or -low group according to the median value of miR-505 expression level. The results showed that low expression of miR-505 was significantly correlated with the tumor size, histological grade, lymph node metastasis and TNM stage (Table 1). Multivariate analysis showed that low expression of miR-505 was an independent unfavorable prognostic factor for the overall survival of HCC patients (Table 2). To evaluate whether the aberrant expression of miR-505 was a potential biomarker for the prognosis of HCC patients, log rank test was performed to analyze the correlation between the expression of miR-505 and the survival of HCC patients. The data showed that lower expression of miR-505 was significantly correlated with the worse prognosis of HCC patients (Figure 1C). These results indicated the potential involvement of miR-505 in the progression of HCC (Table 3).

**Table 3:** Primers used in the present study

Primers	Primer sequence
MiR-505 forward	GCGAGCACCGTCAACACT
MiR-505 Reverse	TGGTGTCGTGGAGTCGGC
U6 Forward	CTCGCTTCGGCAGCACA
U6 Reverse	AACGCTTCACGAATTTGCGT

### Overexpression of miR-505 inhibited the proliferation and induced apoptosis in HCC cells

**Figure 2:**
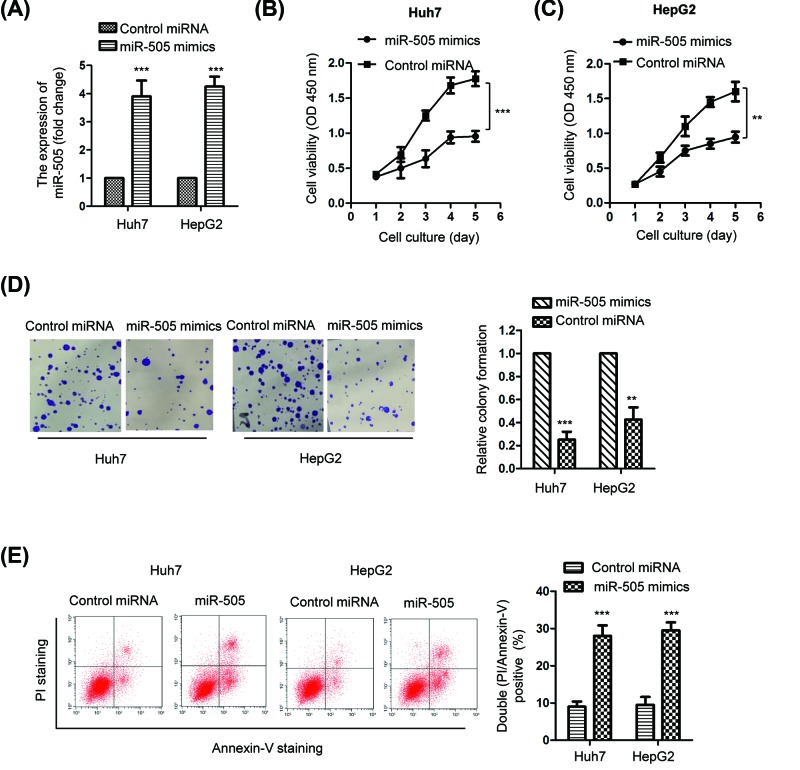
Overexpression of miR-505 inhibited the growth of HCC cells (**A**) HepG2 and Huh7 cells were transfected with miR-505 mimics or control miRNA and the overexpression level of miR-505 was confirmed by RT-qPCR analysis. The expression of U6 RNA was detected as the normalization. (**B**) CCK-8 assay showed that transfection of miR-505 in HepG2 and (**C**) Huh7 cells significantly inhibited the cell proliferation compared with control miRNA transfected cells. (**D**) Overexpression of miR-505 significantly decreased the colony formation of HCC cells. Left, the representative images of the colonies formed by HCC cells expressing miR-505 or control miRNA; Right, the statistical analysis of the colon number. (**E**) FACS analysis showed that the cell apoptosis percentage of HCC cells expressing miR-505 was higher than that of the control group. **P<0.01; ***P<0.001

As the expression of miR-505 was decreased in HCC, to evaluate the function of miR-505 in regulating the proliferation of HCC cells, both HepG2 and Huh7 cells were transfected with miR-505 mimics to up-regulate the expression of miR-505. As shown in Figure 2A, RT-qPCR analysis confirmed the increased expression (∼4.0-fold) of miR-505 in HCC cells with the transfection of miR-505 mimics in comparison with that of the control group. The proliferation of both HepG2 and Huh7 cells expressing miR-505 mimics or control miRNA was determined by the CCK-8 assay. The result showed that overexpressed miR-505 significantly inhibited the proliferation of HCC cells (Figure 2B,C). Additionally, the colony formation assay indicated that ectopic expression of miR-505 markedly suppressed the colony formation of both HepG2 (∼0.23-fold) and Huh7 cells (∼0.40-fold) (Figure 2D). To detect whether miR-505 affected the apoptosis of HCC cells, flow cytometry was performed with HepG2 and Huh7 cells expressing control miRNA or miR-505 mimics. The data showed that highly expressed miR-505 significantly induced the apoptosis of HCC cells (∼10–30%) (Figure 2E).

### IGF-1R was a target of miR-505 in HCC

**Figure 3:**
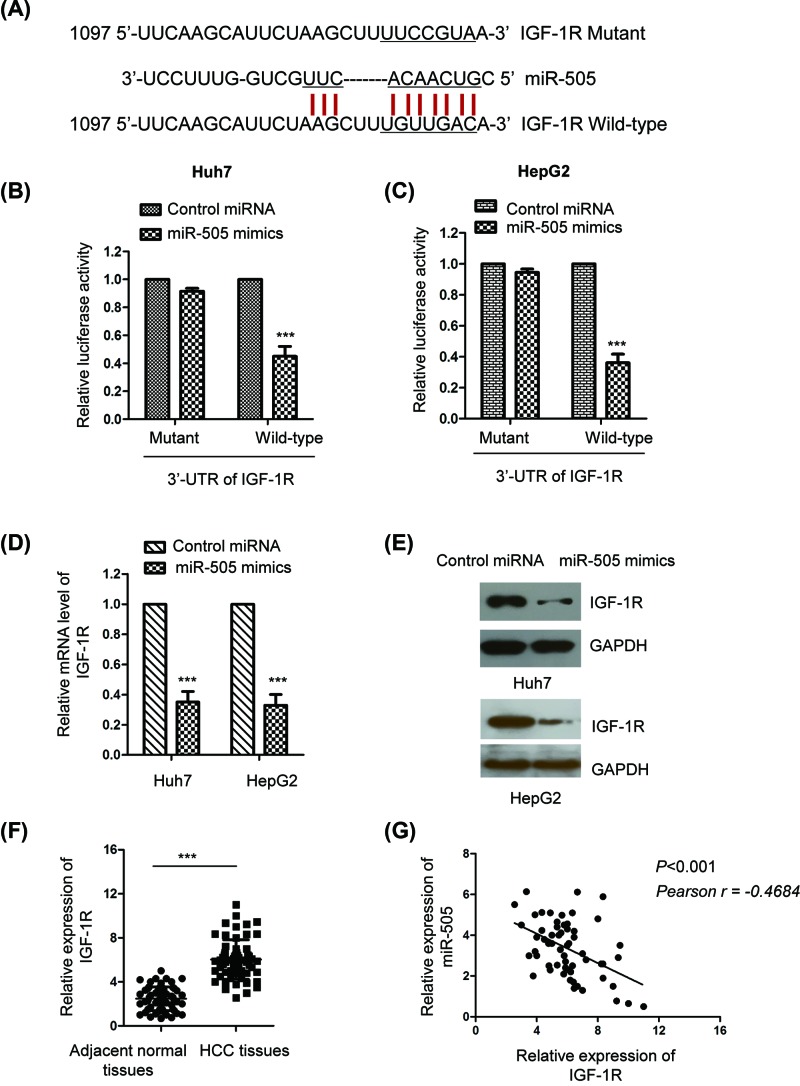
IGF-1R was a down-stream target of miR-505 in HCC cells (**A**) Predicted binding site of miR-505 in the 3′-UTR region of IGF-1R. (**B,C**) HepG2 and Huh7 cells were transfected with the luciferase reporter vector containing wild-type or mutant 3′-UTR of IGF-1R with or without miR-505. The histogram indicated the relative luciferase activity of each group. (**D,E**) The mRNA or protein level of IGF-1R in HCC cells with miR-505 mimics or control miRNA. (**F**) The expression of IGF-1R in paired HCC tissues and adjacent normal tissues was detected by RT-qPCR. (**G**) The correlation between the expression of miR-505 and IGF-1R was analyzed with the Spearman test. ***P<0.001

To explore the underlying mechanism by which miR-505 regulated the growth of HCC cells, the potential targets of miR-505 were predicted using the TargetMiner database. The IGF-1R (NM_000875) was predicted as one of the targets of miR-505 (Supplementary Table S1). The complementary binding between the 3′-UTR sequence of IGF-1R and miR-505 was also predicted with the miRNA.org database http://www.microrna.org/microrna/getGeneForm.do) (Figure 3A). To further verify this prediction, both Huh7 and HepG2 cells were co-transfected with the luciferase reporter vector containing wild-type or mutated 3′-UTR of IGF-1R in the presence of miR-505. As shown in Figure 3B,C, overexpression of miR-505 reduced the luciferase activity harboring wild-type (∼0.43-fold) but not the mutated IGF-1R 3′-UTR in HCC cells. The result indicated the binding of miR-505 with the 3′-UTR of IGF-1R.

To detect whether the binding of miR-505 with the 3′-UTR of IGF-1R affected the expression of IGF-1R, the mRNA level of IGF-1R in HCC cells with the expression of control miRNA or miR-505 mimics was detected by RT-qPCR. As shown in Figure 3D, overexpression of miR-505 significantly decreased the mRNA abundance of IGF-1R (∼0.38-fold) in both HepG2 and Huh7 cells. Moreover, the protein level of IGF-1R was also evaluated by Western blot with the transfection of miR-505. The data showed that up-regulation of miR-505 decreased the protein level of IGF-1R compared with that of control group (Figure 3E). These results suggested miR-505 as a negative regulator of IGF-1R in HCC cells. To further support this conclusion, the expression of IGF-1R in HCC tissues and paired adjacent normal tissues was detected by RT-qPCR. The data indicated the significant up-regulation of IGF-1R in HCC tissues compared with that of adjacent normal tissues (Figure 3F). Spearman test uncovered that the expression of miR-505 was negatively correlated with that of IGF-1R in HCC tissues (Figure 3G). These results supported the conclusion that miR-505 targeted IGF-1R in HCC.

### MiR-505 regulated the IGF-1R/AKT/GLUT1 pathway

**Figure 4:**
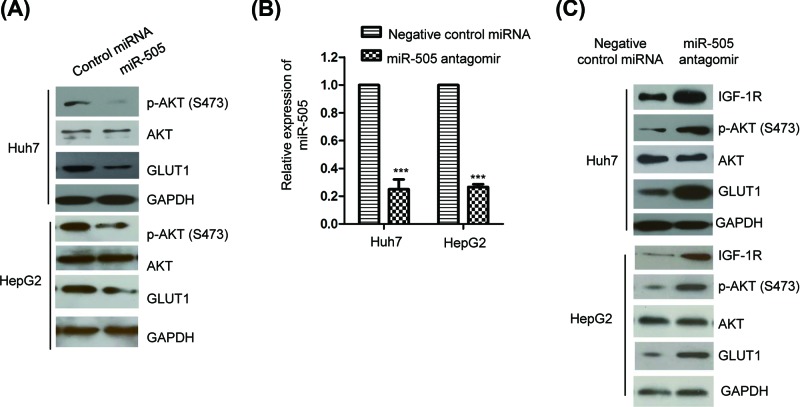
MiR-505 negatively regulated the IGF-1R/AKT/GLUT1 pathway (**A**) Both HepG2 and Huh7 cells were transfected with miR-505 mimics or control miRNA, and the protein level of AKT and GLUT1 was detected with the indicated antibody. (**B**) The endogenous miR-505 was knocked down by inducing miR-505 antagomir into the HCC cells. The decreased expression of miR-505 was checked by the RT-qPCR analysis. (**C**) The protein abundance of IGF-1R, AKT and GLUT1 was detected with HCC cells with depleted miR-505. ***P<0.001

Previous studies indicated that IGF-1R activated AKT and sequentially up-regulated the expression of GLUT1, which promoted the glycolysis of cancer cells. Considering that miR-505 suppressed the expression of IGF-1R, Western blot was performed to examine the levels of AKT and GLUT1 with indicated antibodies. Consistent with the decreased expression of IGF-1R, both the phosphorylation of AKT (S473) and the expression of GLUT1 were reduced in both Huh7 and HepG2 cells with overexpression of miR-505 (Figure 4A). To further confirm these data, the expression of miR-505 was down-regulated (∼0.28-fold) by transfecting miR-505 antagomir into HCC cells. As indicated in Figure 4B, the expression of miR-505 was significantly decreased with the transfection of miR-505 antagomir in Huh7 and HepG2 cells. Western blot analysis showed that down-regulation of miR-505 increased the levels of IGF-1R, the phosphorylation of AKT (S473) and GLUT1 (Figure 4C). These results indicated miR-505 as a novel negative regulator of IGF-1R-AKT-GLUT1 axis in HCC cells.

### MiR-505 modulated the glycolysis of HCC cells

**Figure 5:**
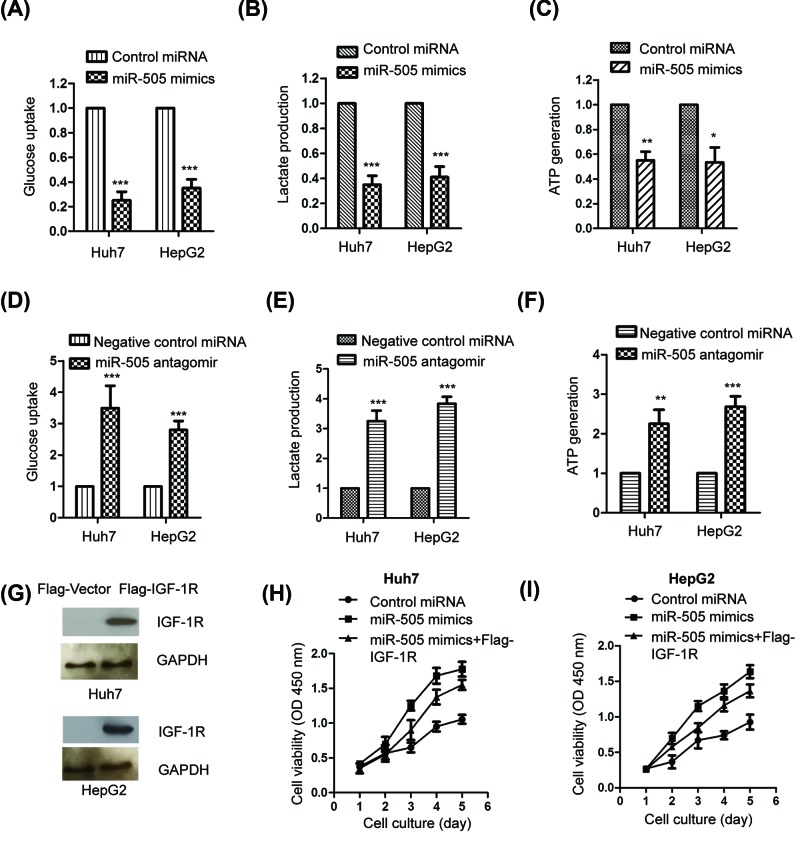
MiR-505 modulated the glycolysis of HCC cells (**A,B**) HepG2 and Huh7 cells were transfected with miR-505 mimics or control miRNA. The glucose consumption and lactate production of the cells were determined. (**C**) The ATP level of the HCC cells expressing the indicated miRNA was detected. (**D,E**) The glucose uptake and lactate production of HCC cells with down-regulated miR-505 was measured. (**F**) HCC cells with depleted miR-505 were lyzed and the ATP concentration was determined. (**G**) Both Huh7 and HepG2 cells were transfected with Flag-vector or Flag-IGF-1R, the expression of Flag tagged IGF-1R was detected by western blot. (**H,I**) Restoration of IGF-1R reversed the inhibitory effect of miR-505 on the proliferation of HCC cells. *P<0.05; **P<0.01; ***P<0.001

The expression of GLUT1 is tightly associated with the glucose uptake of cells. Because overexpression of miR-505 decreased the level of GLUT1, we hypothesized whether aberrant expression of miR-505 influenced the glycolysis of HCC cells. To determine this, the glucose consumption of both Huh7 and HepG2 cells harboring control miRNA or miR-505 mimics was examined. As indicated in Figure 5A, compared with control cells, overexpression of miR-505 significantly reduced the glucose uptake of both HepG2 (∼0.28-fold) and Huh7 cells (∼0.39-fold). Consistently, the lactate level of HCC cells expressing miR-505 was lower (∼0.37-fold) than that of the control cells (Figure 5B). As glucose metabolism is one of the main sources of cellular ATP, the ATP level of HCC cells with the overexpression of miR-505 was quantitated. Overexpressed miR-505 in both HepG2 and Huh7 cells significantly decreased the level of ATP (∼0.55 fold) (Figure 5C), which was consistent with the suppressed glucose uptake and lactate production with miR-505. To further confirm the regulation of miR-505 on the glycolysis of HCC cells, both Huh7 and HepG2 cells were transfected with miR-505 antagomir and the glucose metabolism was investigated. Depletion of miR-505 significantly increased both the glucose uptake and lactate generation of HepG2 (∼3.9-fold) and Huh7 cells (∼2.9-fold) (Figure 5D,E). The ATP level in both Huh7 and HepG2 cells with down-regulation of miR-505 was also increased (∼2.4 and ∼2.8-folds, respectively) (Figure 5F). Collectively, these results suggested miR-505 as a novel negative regulator for the glycolysis of HCC cells. To demonstrate whether the effect of miR-505 on the proliferation of HCC cells was achieved via IGF-1R, Flag tagged IGF-1R was constructed and transfected into both Huh7 and HepG2 cells (Figure 5G). The proliferation of cells expressing miR-505 mimics and Flag-IGF-1R was determined by CCK-8 assay. The results showed that overexpression of miR-505 inhibited the proliferation of HCC cells, however, restoration of IGF-1R significantly reversed the inhibitory effect of miR-505 on the proliferation of HCC cells (Figure 5H,I). These data indicated that IGF-1R played critical roles in mediating the suppressive role of miR-505 in HCC.

## Discussion

HCC is one of the most common malignancies with poor prognosis around the world. Exploring novel factors involved in the development of HCC is quite important for the diagnosis and treatment of HCC. An increasing body of evidence suggested that deregulation of miRNAs plays important roles in the progression of HCC [27,28]. In this study, we found that miR-505 was down-regulated in HCC tissues and cell lines. Decreased miR-505 in HCC was significantly associated with the poorer malignant features of HCC patients, including lymph node metastasis, bigger tumor size and higher TNM stage. These results suggested the potential important role of miR-505 in the progression of HCC.

Recent studies demonstrated that miR-505 was less expressed in cervical cancer and associated with the poor survival of patients [17]. Overexpression of miR-505 suppressed the proliferation and invasion of cervical cancer cell via targeting Frizzled-4 (FZD4) [17]. Additionally, the tumor suppressive function of miR-505 was also confirmed in endometrial carcinoma, which inhibited proliferation and induced apoptosis via suppressing the expression of TGF-α [16]. Reduced expression of miR-505 was also found in hepatoma cell lines including OGY-7703, SMMC-7721 and MHCC97 [19]. Down-regulation of miR-505 suppressed the proliferation, invasion and epithelial–mesenchymal transition of MHCC97 cells via targeting High-Mobility Group Box 1 [19]. In the current study, miR-505 was decreased in HCC tissues and correlated with the advanced progression of HCC patients. Molecular studies indicated that miR-505 targeted and negatively regulated the expression of IGF-1R in HCC cells. IGF-1R was overexpressed in HCC tissues and negatively correlated with that of miR-505. Further investigation was necessary to detect whether the negative modulation of miR-505 on the expression of IGF-1R still occurs in other types of cancers. It is widely accepted that miRNAs usually target many different mRNAs in cells. Previous studies have identified some targets of miR-505 including Frizzled-4 and TGF-α [16,17]. It might be necessary to clarify whether other targets, such as Frizzled-4 and TGF-α, are involved in the suppressive function of miR-505 in HCC.

Cancer cells specifically use aerobic glycolysis for glucose metabolism compared with normal cells. The metabolic reprograming provides energy to facilitate the rapid growth of cancer cells [29]. Therefore, targeting the glycolysis pathway might be a promising strategy to inhibit the tumorigenesis. Increasing evidence uncovered that miRNAs modulated the aerobic glycolysis via targeting the enzymes involved in glycolysis such as GLUT1, G6PD and LDHA [30–33]. IGF-1R is a transmembrane protein with tyrosine kinase activity belonging to the insulin receptor family [34–36]. Up-regulation of IGF-1R has been observed in several cancers, including HCC and renal cell carcinoma [37,38]. It has been reported that IGF-1R interacted with the ligand IGF-1 and stimulated PI3K/AKT signaling pathway [39–41]. Phosphorylation of AKT at S473 regulated the trafficking and activity of GLUT1, which controled the glucose uptake of cancer cell. IKKβ and NF-κB were reported to control the survival of lymphoma cells via AKT-mediated GLUT1 membrane trafficking [42]. In the present study, our results showed that overexpressed miR-505 down-regulated IGF-1R in HCC cells, which consequently inhibited the phosphorylation of AKT and the expression of GLUT1. Consistently, ectopic expression of miR-505 significantly reduced the glucose consumption, lactate production and ATP generation of HCC cells. These data suggested miR-505 as a novel negative regulator of the glycolysis pathway in HCC.

In summary, our results showed that miR-505 was down-regulated in HCC tissues and cell lines. Overexpression of miR-505 suppressed the proliferation of HCC cells. Molecular studies uncover that miR-505 targeted IGF-1R and inhibited the expression of IGF-1R. Decreased expression of IGF-1R inhibited the AKT/GLUT1 pathway and suppressed the glycolysis of HCC cells. These results uncovered the tumor suppressive function of miR-505 in HCC and suggested miR-505 as a promising candidate for treatment of HCC.

## Supporting information

**Supplemental Table S1:** 

## Abbreviations

FBC: fetal bovine serum
FITC: fluorescein isothiocyanate
GLUT: glucose transporter
G6PD: Glucose -6-phosphate dehydrogenase
HCC: hepatocellular carcinoma
HMGB1: High Mobility Group Protein 1
IGF-1R: insulin growth factor receptor
LDHA: Lactate dehydrodenase A
miRNA: microRNA
PI: propidium iodide
RT: room temperature
RT-qPCR: Reverse transcription-quantative polymerase chain reaction
TNM: Tumor, node, metastasis
3′-UTR: 3′-untranslated region
