# Expression of Concern: MiR-505 suppressed the growth of hepatocellular carcinoma cells via targeting IGF-1R

**DOI:** 10.1042/BSR-2018-2442_EOC

**Published:** 2024-11-11

**Keywords:** Hepatocellular carcinoma, IGF-1R, miR-505

The Editorial Office has been made aware of potential issues surrounding the scientific validity of this paper “MiR-505 suppressed the growth of hepatocellular carcinoma cells via targeting IGF-1R” DOI: 10.1042/BSR20182442, hence has issued an expression of concern to notify readers. It has been noted that the flow cytometry scatter graphs in Figure 2E have an unusual appearance that seems to resemble graphs in other papers by different authors. The Editorial Office attempted to contact the authors regarding the concerns raised, however the authors could not be reached.

