# Supplementary material for: MiR-505 suppressed the growth of hepatocellular carcinoma cells via targeting IGF-1R
Source: Biosci Rep. 2019 Jul 2;39(7):BSR20182442. doi: 10.1042/BSR20182442 (PMC6603277; doi:10.1042/BSR20182442)
Supplement: Supplementary file 1 [file bsr20182442_Supp1.pdf]

TargetMiner : Prediction of miRNA Targets

miRNA ID: *hsa-miR-505-3p*
miRNA Sequence: *CGUCAACACUUGCUGGUUUCCU*

mRNA	Chromosome	6mer (count)	6mer (position)	7mer-A1 (count)	7mer-A1 (position)	7mer-m8 (count)	7mer-m8 (position)	8mer (count)	8mer (position)
NM_000259	chr15	4	6014 3963 1547 576	1	4293	2	4752 856	1	484
NM_000430	chr17	4	3542 3222 365 328	1	311	1	2500	1	3177
NM_000809	chr4	6	9228 8469 8274 7333 1272 615	1	8280	3	8180 2808 593	1	7909
NM_000875	chr15	6	6406 6070 5619 4741 4292 1448	1	2703	3	6438 4770 1032	1	1122
NM_001003652	chr18	5	8476 6773 3060 2873 2696	1	6972	2	1476 928	1	5548
NM_001003792	chr3	1	69	1	290	-	-	2	1117 792
NM_001003793	chr3	1	69	1	290	-	-	2	1117 792
NM_001009899	chr3	6	5270 5177 4713 1128 486 475	1	252	1	5413	1	348
NM_001018065	chr9	4	6034 5792 5066 1602	1	4399	1	5825	1	4053
NM_001018066	chr9	4	6034 5792 5066 1602	1	4399	1	5825	1	4053
NM_001024948	chr1	3	3493 1324 1026	-	-	2	3448 2194	1	878
NM_001025077	chr10	7	6679 5354 5337 4917 2143 1800 1146	-	-	2	2825 2332	1	2199
NM_001039618	chr11	7	5607 4898 4133 3812 2212 1369 984	1	712	2	4885 2054	-	-
NM_001040157	chr4	3	886 657 360	-	-	1	2542	2	300 290
NM_001040284	chr16	6	5490 2173 1924 1442 347 110	-	-	1	1220	2	4230 3820
NM_001042555	chr12	3	608 332 277	2	4675 1078	1	2977	1	795
NM_001042599	chr2	3	7324 6744 2836	-	-	1	2207	1	2214
NM_001198	chr6	4	2271 1476 607 598	1	2127	-	-	1	735
NM_001650	chr18	3	4096 2858 814	-	-	1	1020	2	3293 1101
NM_001858	chr6	6	4346 3369 1935 1256 479 366	-	-	2	3614 1287	1	4186
NM_002128	chr13	2	2014 271	-	-	-	-	3	2286 962 424
NM_002577	chr3	1	1325	-	-	1	3094	2	1415 513
NM_002613	chr16	4	4618 3773 1898 748	1	4295	2	5109 4430	2	4853 636
NM_002834	chr12	6	3401 3312 675 573 407 132	-	-	1	697	1	3840
NM_003112	chr7	2	3186 160	1	707	-	-	1	935
NM_003274	chr21	2	2840 1045	-	-	-	-	2	2227 722
NM_003590	chr2	6	3909 2766 1374 1325 330 173	1	876	2	2743 360	1	1391
NM_004028	chr18	3	4096 2858 814	-	-	1	1020	2	3293 1101
NM_004171	chr11	5	6476 6224 6156 1092 624	1	2752	4	9481 8182 3690 1029	1	2760
NM_004685	chr13	1	855	1	2580	1	2766	2	2107 1844
NM_005235	chr2	3	7324 6744 2836	-	-	1	2207	1	2214
NM_005504	chr12	5	6527 5759 5123 4433 1309	1	4807	1	4409	1	4053
NM_005795	chr2	4	2180 732 486 373	-	-	-	-	2	2105 149
NM_005840	chrX	11	7154 6647 5971 5628 5002 2846 2801 2771 2084 2044 1475	-	-	1	5395	-	-
NM_005901	chr18	5	8476 6773 3060 2873 2696	1	6972	2	1476 928	1	5548
NM_006252	chr1	7	7281 4684 2874 2723 1119 1037 488	1	7223	3	6886 6381 5957	-	-
NM_006526	chr20	1	566	-	-	-	-	2	2110 1543
NM_006654	chr12	3	608 332 277	2	4675 1078	1	2977	1	795
NM_007214	chr6	2	1279 99	2	544 444	1	934	1	471
NM_013436	chr2	1	180	-	-	-	-	2	718 655
NM_014423	chr5	7	4764 4752 4499 1938 801 382 170	1	4893	1	5138	1	3649
NM_014820	chr3	7	1599 1551 700 459 332 171 87	1	326	2	395 303	-	-
NM_014953	chr13	5	3652 3507 654 445 151	2	1751 1341	2	1297 1109	-	-
NM_015317	chr2	5	2320 2055 1804 871 860	1	2621	1	1836	1	2393
NM_016009	chr1	4	4047 2983 1099 1025	1	746	-	-	2	4638 1540
NM_016206	chr3	8	8640 7649 6691 3008 2407 808 482 134	2	7873 825	1	7589	2	6726 3243
NM_016217	chr6	5	3567 3335 3117 1360 812	1	2952	1	1130	1	3602
NM_017650	chr7	6	6107 5706 5349 1382 290 140	1	1789	1	798	1	4284
NM_018638	chr12	5	5143 1430 1094 590 574	2	5467 3681	-	-	1	3722
NM_018970	chr7	2	2032 1053	2	2526 374	1	691	1	393
NM_018999	chr10	5	4716 4060 3269 3046 1545	-	-	2	3143 1327	1	4941
NM_020772	chr17	7	8680 2914 2184 1985 1669 717 627	-	-	2	6418 1624	-	-
NM_020917	chr19	2	3610 1207	-	-	2	611 123	2	3311 3050
NM_020948	chr1	3	2607 85 64	-	-	-	-	2	1210 601
NM_021033	chr13	2	2569 2484	2	300 153	-	-	1	2535
NM_021038	chr3	4	3356 2441 2419 2396	-	-	-	-	1	2332
NM_021205	chr1	3	2861 2362 734	1	2322	2	1144 1063	1	688
NM_021629	chr3	3	4835 3145 1326	2	1595 762	-	-	1	4520
NM_022048	chr15	3	6222 5728 264	3	4867 4542 975	4	5737 5630 3940 659	-	-
NM_022166	chr16	4	4213 1242 1081 247	1	5255	1	5919	2	4502 1548
NM_024639	chr6	6	2893 2703 2201 2141 1086 840	2	1833 1121	-	-	1	1159
NM_024947	chr3	9	8391 7082 6515 6447 3365 3122 1674 1257 712	1	7980	4	9407 8254 7503 1759	3	9197 7952 7242
NM_024989	chr2	4	6903 6503 6426 5137	1	4941	2	6240 2380	1	3053
NM_025054	chr8	4	3937 2595 2571 771	-	-	4	1386 966 385 58	-	-
NM_031268	chr16	4	4618 3773 1898 748	1	4295	2	5109 4430	2	4853 636
NM_032424	chr11	2	1045 363	1	1416	-	-	2	1506 1446
NM_032505	chr3	4	2578 2110 1934 829	-	-	-	-	1	1721
NM_032968	chrX	1	4167	2	3890 2768	1	984	1	616
NM_032969	chrX	1	4167	2	3890 2768	1	984	1	616
NM_032973	chrY	1	4159	2	3882 2763	1	984	1	616
NM_033224	chr7	3	6923 2335 1028	2	1284 783	3	7820 6797 1926	1	2803
NM_033389	chr17	5	4501 3419 2997 1778 1303	-	-	1	1334	1	1312
NM_138468	chr2	5	6209 6056 5623 5134 201	-	-	-	-	2	6365 4250
NM_144571	chr4	8	6691 6041 4959 2563 2548 1311 1219 1022	1	4891	2	1202 634	-	-
NM_145307	chr10	5	4707 1871 924 854 249	1	3519	1	3727	1	648
NM_145753	chr3	1	1546	-	-	-	-	2	1682 436
NM_152608	chr1	2	2391 1770	1	1812	-	-	2	383 89
NM_152903	chr13	1	115	2	2137 1860	1	1103	1	2649
NM_153686	chr4	3	2434 1795 490	-	-	1	1922	1	1651
NM_173075	chr4	3	1168 637 525	2	1363 531	-	-	1	547
NM_181521	chr16	6	5699 2351 2080 1790 1113 438	-	-	1	1563	1	1431
NM_182907	chr6	4	2271 1476 607 598	1	2127	-	-	1	735
NM_194314	chr1	5	5100 4405 3943 1828 1341	-	-	-	-	1	830
NM_198273	chr5	3	1264 1044 185	-	-	2	2018 169	1	2354
NM_198859	chr3	11	5016 4764 4223 3617 1910 1435 1399 861 273 186 154	-	-	1	878	-	-
NM_199324	chr4	4	3627 1337 694 359	-	-	1	224	1	2462
NM_205842	chr2	1	180	-	-	-	-	2	718 655
NM_206855	chr6	6	5830 5760 5013 2124 1802 366	-	-	3	6243 1124 660	-	-
NM_207292	chr3	4	3356 2441 2419 2396	-	-	-	-	1	2332
NM_207293	chr3	4	3356 2441 2419 2396	-	-	-	-	1	2332
NM_207294	chr3	4	3356 2441 2419 2396	-	-	-	-	1	2332
NM_207295	chr3	4	3356 2441 2419 2396	-	-	-	-	1	2332
NM_207296	chr3	4	3356 2441 2419 2396	-	-	-	-	1	2332
NM_207297	chr3	4	3381 2466 2444 2421	-	-	-	-	1	2357
NM_207406	chr4	6	6543 6213 4661 2389 2376 1133	1	5454	4	6500 6225 5906 622	1	779
